# Occupational Burnout Symptoms and Its Relationship With Workload and Fear of the SARS-CoV-2 Pandemic Among Hospital Nurses

**DOI:** 10.3389/fpubh.2022.852629

**Published:** 2022-04-27

**Authors:** Marzieh Belji Kangarlou, Farin Fatemi, Fatemeh Paknazar, Alireza Dehdashti

**Affiliations:** ^1^Department of Occupational Health, Semnan University of Medical Sciences, Semnan, Iran; ^2^Research Center of Health Sciences and Technologies, Semnan University of Medical Sciences, Semnan, Iran; ^3^Research Center for Social Determinants of Health, Semnan University of Medical Sciences, Semnan, Iran

**Keywords:** occupational stress, risk factors, hospital nurse, burnout, mental health

## Abstract

**Introduction:**

The pandemic has intensified physical and psychological work demands experienced by nurses in a hospital environment. The purpose of this study was to examine personal and work environmental risk factors associated with occupational burnout among hospital nurses.

**Methods:**

We conducted a cross-sectional from April to November 2020. Data from 831 nurses who worked professionally in four educational hospitals were compiled through survey questionnaires to report the prevalence of burnout, occupational and individual factors. Independent *t*-test and Mann–Whitney test measured the link between the scopes of occupational burnout and risk factors.

**Results:**

About half of the participants indicated moderate symptoms of burnout. The fear of the nurses correlated significantly with emotional exhaustion (*r* = 0.71, *p* = 0.001), depersonalization (*r* = 0.67, *p* = 0.02), and personal accomplishment (*r* = 0.63, *p* = 0.05). Mental demand (*r* = 0.74, *p* = 0.01) and effort at work (*r* = 0.68, 0.001) correlated significantly with emotional exhaustion (*r* = 0.51, *p* = 0.03).

**Conclusion:**

The findings indicated a high prevalence of burnout symptoms, particularly emotional exhaustion, among hospital nursing professionals. Occupational health services should consider burnout as an occupational-related condition and provide interventions to reduce workplace chronic stressors and burnout in hospitals.

## Introduction

With the spread of the COVID-19 pandemic and cases and hospitalizations surging across the countries, the work environment and workload have highly influenced the well-being of hospital nursing professionals (1). Nurses are continuously observing the impacts of coronavirus in hospitals. The exposure of the nurses to daily acute airborne respiratory infections has led to high work-related physical and mental pressures during coronavirus outbreaks (2). According to the World Bank, Iran has 1.9 nurses per 1,000 people, far lower than 5.8 in Italy and 8.3 in the United Stated (3). In Iran, with the onset of coronavirus, many infected patients were admitted to hospitals, and each nurse had to manage, on average, double patients compared to the pre-pandemic situation (4). The nursing professionals' concerns regarding the COVID-19 infection in the hospital, long hours of work, and physical and psychological stress affected their capacity to cope with their workplace stressors in the pandemic era. Moreover, hospital nurses had multiple tasks and responsibilities for patients during the COVID-19 pandemic, so they experienced fatigue, anxiety, and depression (5).

The World Health Organization has classified occupational burnout syndrome as a work-related phenomenon caused by chronic stress (6). The work demands model forms the theoretical concept of individual burnout in this study in which nurses experience burnout as a result of physical and psychological work demands that are higher than their abilities to manage them (7). In general, burnout is a state of physical and mental exhaustion resulting from chronic exposure to occupational stress or physically demanding work-related conditions (8).

Burnout among healthcare providers is a newly emerging challenge that affects healthcare systems and patient safety worldwide (9). Healthcare providers may experience anxiety, irritability, mood swings, insomnia, depression, a sense of failure, and as a result, burnout. Moreover, they may be at risk of severe fatigue in their duties because of alertness and prolonged attention to detail (10).

Previous studies have shown that occupational burnout is linked to stressful workplaces and leads to the poor health of nurses (11, 12). Factors such as job satisfaction, environmental factors in the workplace, workload, daily working hours, and perception of anxiety concerning working conditions can affect occupational burnout among nurses (13). The burnout in nurses can lead to exhaustion, lack of sense of affection for others (depersonalization), and lack of accomplishment and inefficiency. These effects can negatively impact the general health of nurses (14). Moreover, the unfavorable reaction to the clients may be associated with the experience of emotional exhaustion (15).

Increasing physical and mental work demands on the hospital nursing staff have made it crucial to identify the symptoms, and possible risk factors in the workplace, which lead to occupational burnout (16). A better understanding of the association between risk factors and burnout syndrome will help find ways to manage occupational stress, prevent occupational burnout, and thus provide higher quality services, work productivity, and promote community health.

## Aim

The present study examined the associations between burnout symptoms, work-related and individual factors, workload, and fear of the SARS-CoV-2 pandemic among hospital nurses.

### Hypotheses

The research hypothesized that 1—Burnout symptoms are frequent in hospital nurses during pandemic crises. 2—The fear due to COVID-19 in the hospital environment contributes to occupational burnout among nurses. 3—The perception of hospital nurses about workplace stressors has a link with burnout symptoms.

## The Study

### Research Design and Participants

The present cross-sectional study was performed from April to November 2020. The statistical population comprised around 1,200 nursing professionals working in different wards of five teaching hospitals affiliated with the University of Medical Sciences in the province of Semnan in Iran. The participants were the hospital nursing staff, assistants, and technicians from the surgery, dialysis, intensive care, emergency care, cardiac care, internal medicine, gynecology, and pediatric wards. The inclusion criteria were having a nursing work experience of at least 2 years and signing an informed consent form. The nursing staff contracted with COVID-19 or identified with previous psychiatric or mental disorders were excluded. In this study, of all the copies of the questionnaire distributed among nurses in different wards, 831(69.2%) were returned. Also, 31% of the hospital nursing professionals were excluded because they were not eligible or unwilling to respond or failed to complete the questionnaires.

### Data Collection and Instruments

We conducted a paper-based survey and briefed the participants in advance about the project, and sought informed consent for voluntary participation. Three students involved in the study distributed questionnaires and collected data. The questionnaires were in plain Persian language. The first part of the questionnaire was a demographic questionnaire on the nurses' gender, age, marital status, length of nursing work, shift work schedule, education level, and employment status. The second part applied the Iranian version of the Maslach Burnout Inventory- Human Services Survey translated into the Persian language for detecting and assessing the severity of burnout syndrome. It contains 22 items that evaluate burnout with relevant subscales, i.e., emotional exhaustion (EE) (a sense of fatigue and lack of energy), depersonalization (DP) (sense of a negative reaction to the clients and lack of personal accomplishment), and personal accomplishment (PA). The EE subscale has nine statements out of which eight items are related to personal accomplishment, and five items to depersonalization. The frequency of these emotions is measured from 0 (never), 1 (sometimes in a year), 2 (monthly), 3 (a few times a month), 4 (every week), 5 (occasionally in a week), and 6 (every day). Respondents marked their feelings based on the available options.

Higher scores in EE and DP subscales or lower scores in the PA subscale demonstrate a high level of occupational burnout. The following ranges of scores were used to determine the level of burnout as low, moderate, or high. Emotional exhaustion is considered low, moderate, or high if EE, respectively, scores ≤18, 19–26, or ≥27. DP scores of ≤5, 6–9, and ≥10 are considered low, moderate, and high for depersonalization, respectively. PA scores of ≥40, 34–39, and ≤33 demonstrate low, moderate, and high levels of personal accomplishment, respectively. A combination of high score levels for the three burnout domains was attributed to a high level of overall burnout. The internal consistencies were estimated for each three burnout dimensions. The Cronbach's factors were 0.84 for emotional exhaustion, 0.76 for depersonalization, 0.79 for personal accomplishment, and 0.80 for the total scale. The reliability and validity of the burnout instrument in Iran were examined and confirmed by Moalemi et al. who reported a test-retest reliability constant of >0.7 for the three dimensions of burnout (17).

The third part of the questionnaire was used to assess perceived nursing workload by measuring four items of the NASA Task Load Index (TLX). While the original version of the NASA-TLX comprises 6 subscales, an earlier study performed a component analysis to determine the causal patterns between NASA-ionTLX and subscales of the Burnout Inventory. The analysis indicated that two subscales including frustration and performance are similar to emotional and depersonalization components along with measures of burnout, which proposed the four subscales including effort, physical, mental, and temporal demands may be a more direct determinant of task load (18). The items were as follows: How much thinking do you require to perform a task? How much is the intensity of physical activity at work? How much time pressure do you feel at work? How hard do you have to work? The instrument assessed workload on five 7-point scales. The overall workload scale ranging from 0 to 100 was obtained by combining the six scales. The workload scale was incremented as “low”, “medium”, and “high” estimates. The Cronbach's alpha was estimated at 0.71 for the nursing workload in this study.

The fourth part of the questionnaire assessed the scale of fear perceived by the nurses during the spread of SARS-CoV-2. The fear scale consisted of four items, which were adapted from the perceived threat of the COVID-19 scale (16): Fear of contracting coronavirus infection, spreading coronavirus to family (worry about taking it home to family), afraid for the future, and fear of death. The nurses were requested to answer a four-point Likert scale from “no” fear to “high” level of fear. The scores were from 4 to 16. The total score was classified as negligible (≤8), moderate (9–12), and high fear of the pandemic (13–16). The internal validity was assessed by three health professionals. In this study, Cronbach's alpha was 0.84.

### Statistical Methods

After the participants completed the questionnaires, the collected data were first scored based on the instructions for the burnout symptom instrument and then analyzed in SPSS 22. This study used statistical tests of percentage, frequency, mean, SD, maximum, and minimum to describe personal and occupational factors, including age, sex, marital status, employment, work hours, and experience.

This study applied an independent *t*-test and Mann–Whitney U test to measure the link between the scopes of occupational burnout and personal factors such as sex and marital status. Kruskal–Wallis and one-way ANOVA examined the relationship between occupational burnout and independent risk factors. Spearman's test compared demographic variables with the scopes of occupational burnout. Pearson correlation test assessed the different dimensions and their relations to occupational burnout and task load dimensions. The analysis used frequency percentages to compare occupational burnout in different hospital wards. The significance level was *p* ≤ 0.05.

### Ethical Considerations

We conducted this study according to the rules proposed by the Research Ethics Review Committee of the University Board. Following necessary coordination with the authorities of hospitals, the eligible nurses were enrolled. The nurses were briefed about the study and asked to answer all the questions in the questionnaires. The participation in the study was voluntary and with informed consent. Moreover, we ensured the nurses of data privacy and relevant ethical aspects. The research team coded hospitals, wards, and respondents in the questionnaire to protect their anonymity and confidentiality.

## Results

### Individual and Occupational Characteristics of the Participants

Of the 831 nurses, 602 (72.5%) were women and 229 (27.5%) were men. The means and standard deviations of the nurses' age were 34.72 ± 0.7 (ranging 23–49 years), and work experience was 11.19 ± 0.6 (range: 2–29 years). Most of the nurses (93.1%) had a master's degree. Most nurses had received training for safe work procedures in the hospital environment. Over half of the hospital nurses believed that safety and health were acceptable in the work environment. Data related to the characteristics of the hospital nurses who participated in this research are given in Table 1.

**Table 1:** Characteristics of the hospital nurses at studied teaching hospitals (*n* = 831).

**Variables**	**Frequency (%)**
**Gender**	
Female	602 (72.5)
Male	229 (27.5)
**Marital status**	
Single	190 (22.9)
Married	641 (77.1)
**Employment status**	
Permanent	545 (65.6)
Temporary	127 (15.3)
Informal	159 (19.1)
**Working hours**	
7 h	406 (48.9)
8 h	266 (32.1)
10 h	69 (8.4)
12 h	90 (10.7)
**Training on safe work procedures**	
Yes	699 (84.2)
No	132 (15.8)
**Evaluation of safety and health at work environment**	
Acceptable	346 (41.6)
Unacceptable	485 (58.4)

### Occupational Burnout, Task Load, and Feeling of Fear in Hospital Nurses

Table 2 demonstrates the burnout symptoms, workload, and fear perceived by the nurses during the pandemic.

**Table 2:** Burnout symptoms and task load items and fear felt by nurses during work under COVID-19 at studied teaching hospitals (*n* = 831).

**Variables**	***N* (%)**	**Mean (SD)**
Emotional exhaustion Low Moderate High	456 (54.9) 326 (39.1) 49 (6)	26.30 (11.02)
Depersonalization Low Moderate High	615 (74.02) 143 (17.26) 73 (8.70)	10.90 (4.31)
Personal accomplishment Low Moderate High	115 (13.9) 309 (37.3) 407 (49.05)	27.10 (9.28)
Mental demand Low Moderate High	45 (5.41) 489 (58.84) 297 (35.74)	81.30 (22.18)
Physical demand Low Moderate High	96 (11.55) 523 (62.94) 212 (25.51)	51.62 (24.30)
Temporal demand Low Moderate High	34 (4.09) 462 (55.59) 335 (40.31)	79.86 (26.14)
Effort Low Moderate High	72 (8.66) 519 (62.45) 240 (28.88)	84.35 (24.20)
Fear of Covid-19 No or mild Moderate High	32 (3.85) 102 (12.27) 697 (83.87)	14.17 (3.57)

The hospital nurses reported high levels of emotion exhaustion (mean = 37.09, SD = ±0.8), depersonalization (mean = 24.9, SD = ± 0.3), and personal accomplishment (mean = 24.9, SD = ± 0.3). Overall, the nurses had a high task load during the pandemic. The analysis of our data showed that over half of the nurses felt moderate to high time pressure, physical and mental demands, and efforts because of their tasks. Most nurses perceived the fear of COVID-19 threat to their well-being at work (83.87%). The findings showed that 39.1% of the nurses experienced moderate emotional exhaustion, 8.7% had a high score of depersonalization, and 13.9% had a low score of personal accomplishment.

Table 3 shows the odds of having a high level of overall burnout in the nurses working in hospital wards. Working in different hospital department settings was not significantly associated with the high level of burnout symptoms.

**Table 3:** Association of working in hospital wards with high level of burnout symptoms in studied hospitals.

**Type of Wards**	**Burnout symptoms at high level %**	**Unadjusted odd ratio (95% confidence interval)**	^*^Adjusted odd ratio (95% confidence interval)
Surgery	1.5	Reference	Reference
Dialysis	4.6	0.38(0.22–1.23)	0.54(0.3–1.30)
Intensive care	6.3	0.69(0.59–2.45)	1.07(1.05–2.60)
Cardiac care	7.1	1.23(0.37–8.02)	1.03(0.98–1.60)
Pediatric	7.6	1.09(1.03–4.56)	1.71(1.04–2.40)
Internal medicine	19.9	1.21(0.34–5.43)	1.70(1.44–3.34)
Gynecology	20.9	2.03(1.46–5.92)	1.52(1.06–3.91)
Emergency	32.3	2.18(1.03–7.48)	1.50(1.60–5.70)

* *Adjusted estimates are based on participants responded for all variables*.

### Differences in Burnout, Task Load, and Fear Levels in Terms of Nurses' Characteristics and Occupational Factors

Table 4 presents the mean, standard deviation, and variances in the scores of burnout domains, task load, and fear of pandemic between independent variables of personal and occupational subgroups. The analysis of our data showed that the levels of overall burnout were not statistically different between subgroups of sex and marital status, and employment type. However, significant statistical differences in overall burnout were observed between subgroups of age (*p* = 0.03), hours of performing work (*p* = 0.01), and length of work experience (*p* = 0.01). Depersonalization levels were significant between female and male participants (*p* = 0.01). The degree of threat caused by the pandemic was statistically different between subgroups of variables of sex (*p* = 0.001), marital status (*p* = 0.01), age (*p* = 0.02), length of work experience (*p* = 0.005), type of employment (*p* = 0.01), and hours of performing work in a shift (*p* = 0.01).

**Table 4:** Differences in the dimensions of burnout, overall burnout, task load, and fear of COVID-19 in categorized subgroups in terms of individual and work characteristics in hospital nurses.

**Variables**	**Emotional exhaustion**		**Depersonalization**		**Personal accomplishment**		**Overall burnout**		**Task load**		**Fear**	
	**Mean** **±SD**	***p*** **-value**	**Mean** **±SD**	***p*** **-value**	**Mean** **±SD**	***p*** **-value**	**Mean** **±SD**	***p*** **-value**	**Mean** **±SD**	***p*** **-value**	**Mean** **±SD**	***p*** **-value**
**Sex**
Female	29.70 ± 12.01	0.4	11.5 ± 0.8	0.01	27.9 ± 0.7	0.2	88.06 ± 2.1	0.3	73.1 ± 18.2	0.1	15.3 ± 2.6	0.001
Male	28.05 ± 9.16		9.4 ± 0.4		27.3 ± 1.1		90.1 ± 1.7		77.6 ± 24.5		12.2 ± 4.9	
**Marital Status**
Single	28.3 ± 1.7	0.3	12.2 ± 1.03	0.1	28.3 ± 1.3	0.5	89.8 ± 1.1	0.7	68.7 ± 16.8	0.1	12.4 ± 3.3	0.01
Married	26.7 ± 0.9		10.3 ± 0.3		27.5 ± 0.6		89.5 ± 0.6		72.1 ± 14.3		15.3 ± 2.9	
**Age**
23–30	27.3 ± 7.3	0.1	13.8 ± 3.8	0.02	25.6 ± 6.2	*0.01	86.9 ± 13.02	0.03	65.1 ± 17.3	0.3	14.2 ± 2.5	0.02
31–40	27.9 ± 8.7		10.4 ± 3.9		27.9 ± 7.3		91.4 ± 17.2		70.3 ± 22.9		14.8 ± 2.2	
41–49	29.5 ± 4.8		8.1 ± 3.02		31.8 ± 6.8		93.9 ± 12.3		73.22 ± 25.7		15.1 ± 2.4	
**Length of work Experience**
2–9	24.7 ± 7.9	0.1	14.7 ± 7.9	0.04	24.2 ± 5.9	0.02	70.8 ± 14.6	0.01	75.3 ± 20.7	0.4	14.3 ± 3.1	0.005
10–19	27.6 ± 9.6		17.6 ± 9.6		28.1 ± 3.9		85.6 ± 14.5		71.4 ± 16.2		15.1 ± 4.1	
20–29	24.7 ± 8.4		14.7 ± 8.4		30.7 ± 4.2		92.4 ± 8.5		71.9 ± 18.8		14.9 ± 3.1	
**Employment Status**
Permanent	26.4 ± 1.07	0.5	16.4 ± 1.07	0.7	28.7 ± 0.7	0.3	90.5 ± 0.7	0.3	73.4 ± 15.1	0.3	14.8 ± 2.8	0.01
Temporary	39.0 ± 2.09		9 ± 2.09		27.1 ± 1.5		91.3 ± 1.4		74.1 ± 16.9		12.3 ± 3.2	
Informal	37.8 ± 1. 7		11.8 ± 1.7		24.7 ± 1.4		85.3 ± 1.3		68.4 ± 20.7		13.1 ± 4.1	
**Working hours**
7 h	28.03 ± 8.9	0.3	15.7 ± 4.09	0.04	28.5 ± 6.9	0.2	92.3 ± 14.9	0.01	70.4 ± 13.1	0.2	13.1 ± 3.6	0.01
8 h	25.4 11.08		13.4 ± 4.08		26.6 ± 7.5		85.4 ± 18.6		70.4 ± 16.3		13.9 ± 4.5	
10 h	27.2 ± 8.2		15 ± 3.7		29.4 ± 5		91.7 ± 10.8		72.9 ± 16.8		14.5 ± 3.7	
12 h	27.7 ± 9.2		17.7 ± 9.2		26.2 ± 6.8		89.4 ± 16.9		72.1 ± 15.7		14.4 ± 4.3	

### Associations Among Burnout Dimensions, Task Load, and Fear of SARS-CoV-2

Table 5 gives the correlations between dimensions of burnout, task load, and feelings of fear perceived among hospital nurses during the SARS-CoV-2 pandemic. A considerable degree of correlation existed among the three main variables of emotional exhaustion, depersonalization, and personal achievement. Emotional exhaustion was directly correlated with personal achievement, as an increase in emotional exhaustion increased PA (*r* = 0.19, *p* = 0.001).

**Table 5:** Correlations (*p*-values) between emotional exhaustion, depersonalization, and personal accomplishment among studied hospital nurses.

**Variables**	**1**	**2**	**3**	**4**	**5**	**6**	**7**	**8**
1. Burnout: Emotional exhaustion	1							
2. Burnout: Depersonalization	0.12 (0.01)	1						
3. Burnout: Personal accomplishment	0.13 (0.06)	0.19 (0.01)	1					
4. Mental demand	0.74 (0.01)	0.51 (0.03)	0.67 (0.002)	1				
5. Physical demand	0.22 (0.01)	0.20 (0.19)	0.27 (0.02)	0.29 (0.002)	1			
6. Temporal demand	0.28 (0.03)	0.22 (0.06)	0.39 (0.04)	0.61 (0.01)	0.32 (0.007)	1		
7. Effort	0.68 (0.001)	0.51 (0.03)	0.58 (0.01)	0.49 (0.04)	0.38 (0.02)	0.37 (0.003)	1	
8. Fear of Covid-19	0.71 (0.001)	0.67 (0.02)	0.63 (0.05)	0.26 (0.07)	0.21 (0.01)	0.28 (0.05)	0.24 (0.01)	1

The analyses indicated that emotional exhaustion highly correlated with the nurses' effort (*r* = 0.68, *p* = 0.001). Depersonalization had higher correlation with mental demand (*r* = 0.51, *p* = 0.03), effort (*r* = 0.51). Personal achievement had higher correlation with mental demand (*r* = 0.67), effort (0.58). The fear of SARS-CoV-2 perceived by nurses associated significantly with emotional exhaustion (*r* = 0.65, *p* = 0.03), depersonalization (*r* = 0.67, *p* = 0.02), and personal accomplishment (*r* = 0.63, *p* = 0.05).

## Discussion

This work studied the prevalence of burnout and relationships between individual characteristics and occupational risk factors and burnout among Iranian hospital nurses during the SARS-CoV-2 pandemic. Our results showed a relatively high prevalence of emotional state of energy depletion and exhaustion, increased emotional distance from the nursing profession, and reduced professional efficacy in the nursing profession during the COVID-19 pandemic. We found that workload stressors perceived under the status of novel coronavirus contributed to burnout in hospital nurses. Our results revealed that years at work, age, and length of working hours were associated with burnout, but the type of employment had no significant association with burnout dimensions.

In our study, 54% of the nurses scored moderate level of emotional exhaustion, 8.7% scored high for depersonalization, and 13.9 % scored low on the accomplishment variable. Additionally, we found a higher frequency of burnout among hospital nurses in this study as compared to the studies before the coronavirus pandemic in Iran (19). The COVID-19 pandemic and workforce shortages that imposed extended hours of work for many nurses may explain the increased rate of burnout symptoms. Similar results reported high rates of burnout among health workers in the United Kingdom and Poland (20).

However, various studies reported different scores of burnout dimensions in the nursing profession (21, 22). In this study, the average scores of emotional fatigue were higher than other burnout dimensions. This result is in agreement with a Spanish research on burnout prevalence which reported emotional exhaustion among hospital nurses (16).

We found that all three dimensions of burnout were more frequent among nurses offering acute services in emergency wards. A previous study demonstrated a link between nurses' unpleasant relationships with coworkers and supervisors in emergency services and emotional exhaustion (23). Furthermore, our study found a link between a higher level of burnout symptoms and a higher level of work strains. Some studies have already suggested that nurses who care for severely ill patients with high job demands in terms of workload and work pressure had experienced more feelings of frustration, which may initiate burnout symptoms (3, 8, 24).

Our results showed that working in populated hospital settings and continuously wearing protective devices during the working period may have increased the risk of burnout. These findings are consistent with a previous study, which compared workloads across professions and reported high burnout incidence because of excessive workloads and high mental demands (25, 26).

We found a moderate level of depersonalization correlated with age, gender, work hours, and experience, which is similar to the results of prior studies (27, 28). Our study revealed a higher rate of work-related burnout among nurses in emergency units than nurses in other units. A previous study reported a higher prevalence of burnout among emergency nurses might relate to work circumstances, particularly excessive extended working hours, psychological workload, and organizational factors (29–31).

This study found a link between years of work experience and individual accomplishment and depersonalization. We also found that nurses with older age may be related to higher personal accomplishment levels. Previous studies reported different results concerning the role of age in burnout symptoms. In a previous study on nurses, Elbarazi et al. (9) found that personal accomplishment improved with increasing age (9). Inversely, Lawn et al. (33) reported that older nurses had lower personal accomplishments but found no significant association between age and working years with emotional exhaustion or depersonalization (32). Furthermore, studies by Elbarazi et al. on emergency health care workers and medical doctors have not found a relationship between age and years of employment with burnout (9).

This study showed that gender and marital status have no statistical association with work-related burnout. These results are consistent with the findings of an earlier study on clinical nurses (33). However, some studies have claimed the association between age and gender with occupational burnout (34, 35).

In this study, our results pointed to no significant association between employment status in the forms of permanent, temporary, and informal work with burnout syndromes. However, a previous study before the onset of COVID-19 has reported an association between precarious employment and health (36). The probable explanation for our result that employment status was not a significant factor in burnout among nursing professionals would be the high work environment stress experienced under SARS-CoV-2.

In this study, extended working hours had a link to burnout. Nurses with longer hours of work were more likely to experience the depersonalization aspect of burnout, which is in line with a recent study that showed health care workers who worked continuously in an extended 12-h shift, experienced more burnout and human error during their tasks (37). Prior research on hospital nurses reported that extended working hours might increase job dissatisfaction and burnout compared to a standard 8-h shift work. Besides, nurses with extended working hours had lower work performance (7).

This study shows that nurses are particularly susceptible to burnout because of the workload, environmental pressure, and chaos they confront. In our study, the mental demand for nursing tasks contributed to a higher level of depersonalization, which is similar to previous reports (10, 38). Furthermore, our study showed that the level of experienced depersonalization might explain individual accomplishment (13).

### Study Limitations

This study had some limitations that might influence when inferring the findings. First, despite collecting data on individual and work-related characteristics, this study did not include other variables that might relate to burnout, work-family conflict, and organizational factors. Future studies may examine a broader range of variables inside and outside the work environment. Second, although our study included a representative sample of hospital nurses, we applied a cross-sectional design, and thus this study cannot conclude causal relationships. Finally, this study used a self-report to obtain data for describing workload, mental, and physical burnout, which resulted in possible responses nurses believed to be desirable for their well-being. However, we asked nurses to respond to the questionnaire anonymously to improve a more reliable response.

## Conclusion

This study indicates that the work of nursing professionals under the high stress of COVID-19 could have a substantial impact on the prevalence of burnout dimensions. Increased work demands and patterns imposed on hospital nurses highlight links between risk factors and significant effects on nurses' health. Stress, fear, and overwork caused by the pandemic in the hospital environment pushed nurses beyond their capacity to cope with prolonged physical and mental strain. These factors may lead to more issues, such as anxiety, depression, and chronic fatigue. Workplace well-being units need to boost nurses' level of resilience by safely responding to stress.

Perceived high level of fear and extended working hours following the spread of COVID-19 contributed to increasing occupational burnout among nurses. Burnout, in turn, has affected their family life and their ability to provide services to patients, which rationalized the workplace well-being units to take interventions. Interventions should focus on the emotional support of hospital nurses to enable them to manage their behavior and responses to work demand and hazardous environmental conditions. This study indicated the relatively high frequency of three dimensions of burnout, particularly symptoms of emotional exhaustion among hospital nurses. Workplace well-being units should provide occupational health services to reduce stressful work environment conditions in hospital settings. Future studies on workplace burnout should focus on actions that may prevent symptoms in nursing professions.

## Data Availability Statement

The raw data supporting the conclusions of this article will be made available by the authors, without undue reservation.

## Ethics Statement

This study was performed in line with the principles of the Declaration of Helsinki. The research protocol was approved by the Research Ethics Committee of Medical Sciences University (IR.SEMUMS.REC.1400.176).

## Author Contributions

AD, MB, FF, FP contributed to the study material preparation, conceptualization, methodology, data collection, analysis, interpretation, and writing, reviewing and revising the manuscript. All authors reviewed and commented on previous versions of the manuscript. All authors read and approved the final manuscript. All authors contributed to the study conception, design, and investigation.

## Conflict of Interest

The authors declare that the research was conducted in the absence of any commercial or financial relationships that could be construed as a potential conflict of interest.

## Publisher's Note

All claims expressed in this article are solely those of the authors and do not necessarily represent those of their affiliated organizations, or those of the publisher, the editors and the reviewers. Any product that may be evaluated in this article, or claim that may be made by its manufacturer, is not guaranteed or endorsed by the publisher.
